# Deciphering regeneration through non-model animals: A century of experiments on cephalopod mollusks and an outlook at the future

**DOI:** 10.3389/fcell.2022.1072382

**Published:** 2023-01-09

**Authors:** Fabio De Sio, Pamela Imperadore

**Affiliations:** ^1^ Heinrich Heine Universität, Institut für Geschichte, Theorie und Ethik der Medizin, Centre for Health and Society, Medizinische Fakultät, Düsseldorf, Germany; ^2^ Department of Biology and Evolution of Marine Organisms, Stazione Zoologica Anton Dohrn, Napoli, Italy; ^3^ Association for Cephalopod Research—CephRes, Napoli, Italy

**Keywords:** history of science, invertebrates, octopus, regeneration, cellular and molecular pathways, arm, hectocotylus, pallial nerve

## Abstract

The advent of marine stations in the last quarter of the 19th Century has given biologists the possibility of observing and experimenting upon myriad marine organisms. Among them, cephalopod mollusks have attracted great attention from the onset, thanks to their remarkable adaptability to captivity and a great number of biologically unique features including a sophisticate behavioral repertoire, remarkable body patterning capacities under direct neural control and the complexity of nervous system rivalling vertebrates. Surprisingly, the capacity to regenerate tissues and complex structures, such as appendages, albeit been known for centuries, has been understudied over the decades. Here, we will first review the limited in number, but fundamental studies on the subject published between 1920 and 1970 and discuss what they added to our knowledge of regeneration as a biological phenomenon. We will also speculate on how these relate to their epistemic and disciplinary context, setting the base for the study of regeneration in the taxon. We will then frame the peripherality of cephalopods in regeneration studies in relation with their experimental accessibility, and in comparison, with established models, either simpler (such as planarians), or more promising in terms of translation (urodeles). Last, we will explore the potential and growing relevance of cephalopods as prospective models of regeneration today, in the light of the novel opportunities provided by technological and methodological advances, to reconsider old problems and explore new ones. The recent development of cutting-edge technologies made available for cephalopods, like genome editing, is allowing for a number of important findings and opening the way toward new promising avenues. The contribution offered by cephalopods will increase our knowledge on regenerative mechanisms through cross-species comparison and will lead to a better understanding of the complex cellular and molecular machinery involved, shedding a light on the common pathways but also on the novel strategies different taxa evolved to promote regeneration of tissues and organs. Through the dialogue between biological/experimental and historical/contextual perspectives, this article will stimulate a discussion around the changing relations between availability of animal models and their specificity, technical and methodological developments and scientific trends in contemporary biology and medicine.

## Introduction

The history of the observations of regeneration in cephalopods is centuries-long. The iconographic record suggests that the encounter with octopuses (especially) with damaged arms at different stages of regrowth was not exceptional (see for example Figure 4 in Nakajima et al., 2018). Nevertheless, the first published observations about cephalopod arms regeneration date back to the mid-XIX Century (Vérany, 1851; Vérany and Vogt, 1852; Steenstrup, 1856a), and, even then, only in connection with a specific natural-historical problem: the distinction between sexes.

The first experimental study on cephalopod regeneration (Lange, 1920) only appeared about 60 years later, when marine stations made the wealth of marine life-forms accessible to zoologists and comparative physiologists.

In the following century, both regeneration and cephalopods became objects of intense experimental work. Yet, despite repeated confirmation of cephalopod regenerative capacities, their employment in this field remained scant (for a review see Imperadore and Fiorito, 2018). Indeed, one does not need all the fingers of both hands to count them all. Moreover, they are either one-off studies within a larger comparative framework, or the results of occasional observations, or, finally, largely unsuccessful attempts at starting a sustained research endeavour (until the very present, at least). In this connection, it is perhaps worth mentioning that the four most complete and in-depth studies of regeneration in cephalopods in the XX Century are doctoral dissertations (Lange, 1920; Féral, 1977; Imperadore, 2017; Baldascino, 2019).

This article addresses this very question: how is it that, despite the growing popularity and availability of cephalopods in the laboratory and the intriguing examples of regeneration they offer, they have remained so irreducibly peripheral to this field of research?

We will approach the problem through an analysis of the earlier works, their contextualisation within the experimental cultures within which they were born, and their specific framing in the changing epistemic focuses on regeneration as a phenomenon, a research field, and a biomedical problem.

## Early experimental studies

### The hectocotylus—A natural-historical prologue

**FIGURE 1:**
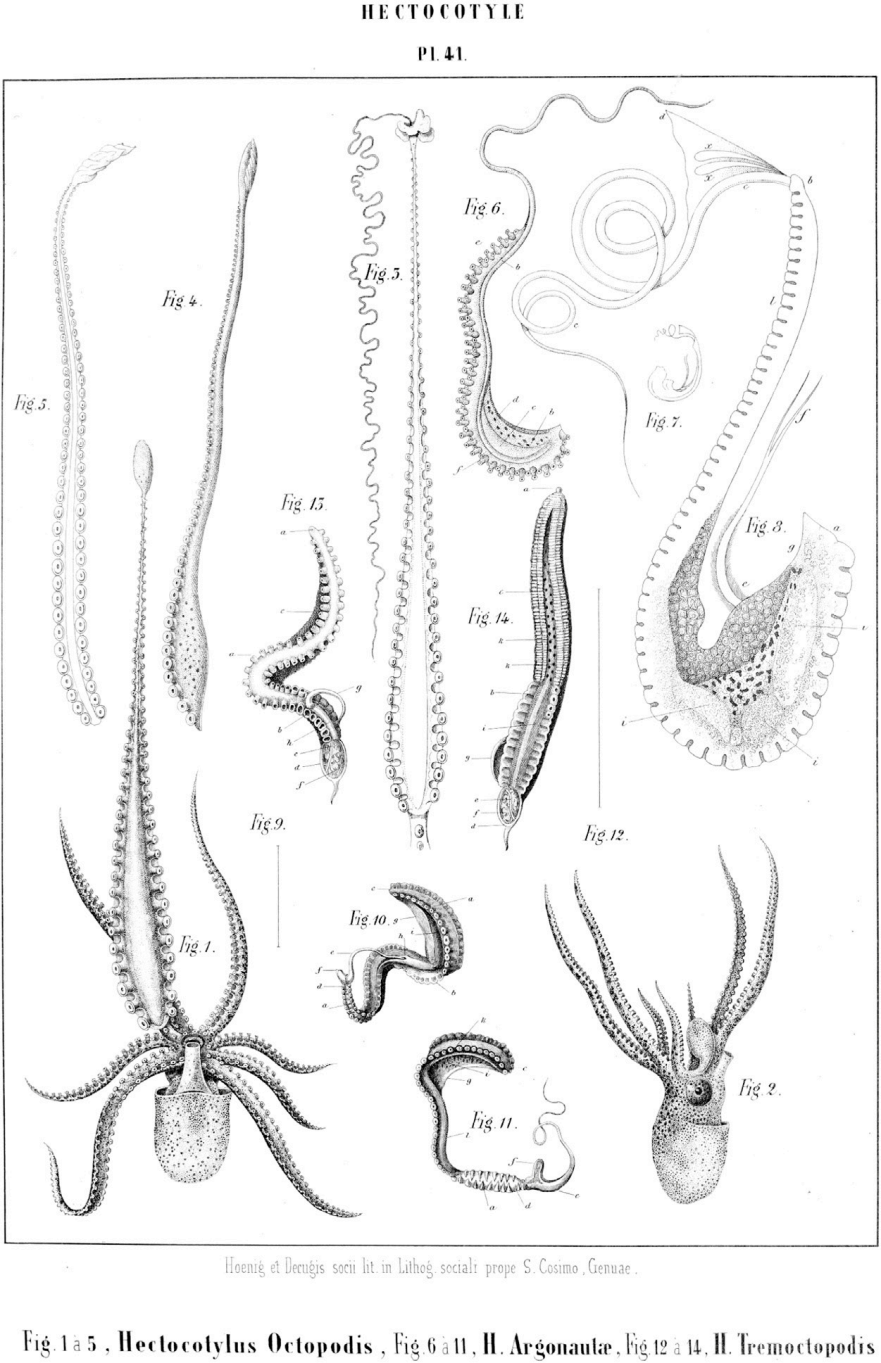
Hectocotyli of cephalopods. 1-5. *Octopus vulgaris*; 6-11. *Argonauta argo*; 12-14. *Tremoctopus violaceus* (Vérany, 1851, table 41. Out of copyright).

In 1856, the Danish naturalist Johannes Japetus Smith Steenstrup, published, in the Memoires of the Royal Academy of Sciences and Letters of Denmark, a detailed study of an “essential deviation from the symmetrical structure” in the octopods *Argonauta argo* and *Tremoctopus violaceus* (Steenstrup, 1856a). The identified structure went by the arcane name of “Hectocotylus” (hundred-fold tube) and, since its first description by Stefano Delle Chiaje (who had christened it *Trichocephalus acetabularis*, “hair-sized head with suckers”, Delle Chiaje, 1825: p. 225ff), it had undergone several changes of identity. Delle Chiaje (1825) and Cuvier (1829) described it as a parasitic worm, endowed with great liveliness and motility, as well as a staggering resemblance to an octopod arm. To the zoologist Rudolf Kölliker and his colleague, the comparative anatomist Carl von Siebold, the hectocotylus was instead the (never observed before) male form of three octopus species (*Argonauta*, *Tremoctopus* and *Eledone rugosa*), on account of its complex internal structure (Kölliker, 1846; Kölliker, 1849);. In advancing this hypothesis, they both relied on personal examinations on *Tremoctopus* specimens and on some earlier observations by the French zoologist Jeannette Villepreux-Power on argonauts (Villepreux-Powers, 1837). Although Siebold was confident enough to include this explanation in his influential manual of comparative anatomy (Siebold, 1848: p. 363ff), his optimism was to prove hasty: soon after, the Würzburg anatomist Heinrich Müller, and the Italian amateur naturalist Jean-Baptiste Vérany, through a series of well-aimed (and lucky) observations, put the matter to rest. Vérany had indeed engaged in a census of the marine species of the Mediterranean coast since the early 1830s. Between 1847 and 1851, he condensed the results in the first part of his *Mollusques Méditerraneens*, devoted to cephalopods (Vérany, 1851). The last entry of this census was on the *Hectocotylus* (p. 126), and contained an abridged history of the controversy, followed by his suggested solution: the *Hectocotylus octopodis*, proposed by Cuvier, was nothing else than the deciduous, regenerating sexual arm of the octopus, while this was not the case for *Argonauta* and *Tremoctopus* (Figure 1). However, in 1851, a short note by Müller announced the identification of male *Argonauta*, described as much smaller in size than female specimens, and the recognition of *Hectocotylus argonautae* as part of the animal (Müller, 1851). Thus, the nature of the hectocotylus as detachable sexual arm was confirmed for argonauts and, by inference, for *T. violaceus*, the male of which was still unknown.

The following year, Vérany and the Swiss zoologist Carl Vogt, published a lengthy account of the anatomy and behaviour of the hectocotylus (Vérany and Vogt, 1852), based on the observation of living animals and of the fresh specimens obtained from anglers in Nice and Genoa. Most of these studies were on *O. carenae* Vérany, 1839 (accepted name *Ocythoe tuberculata* Rafinesque, 1814), which lent itself especially well to *in vivo* anatomical examination due to the transparency of its tissues, allowing observation of the structure of the hectocotylus as part of the living animal (Vérany and Vogt, 1852: p. 176). Through regular visits to the fish markets the two were also able to secure a few specimens of argonauts and *T. violaceus*, for comparison, but could not find any males and thus relied on personal communication by Müller on the argonaut, and analogical reasoning for the other species. In 1853, also Müller completed his study, which found place in Kölliker and Siebold’s *Zeitschrift für Wissenschaftliche Zoologie* (Müller, 1853).

The three scholars finally put order in the puzzling series of observations and interpretations of the previous decades providing a thorough description of the main features of the organ, including the constancy and species-specificity of its position, the greater ease with which it could be removed from its basis, as opposed to the other arms (Vérany and Vogt, 1852: p. 155) and the persisting liveliness of the separated hectocotyli. These last two characteristics found an explanation (at least in the case of the argonauts) in the special challenges posed by copulation in species with such a remarkable sexual dimorphism. Both works also proposed speculations about the regeneration potential of the hectocotyli, by implication from the “well known fact” (Vérany, 1851) that it was difficult to find any living octopod without at least one regenerating arm.

These conclusions had immediate diffusion among naturalists, through translations (Henfrey and Huxley, 1853) and textbook summaries (Owen, 1855: p. 630-632).

Steenstrup’s 1856 contribution (Steenstrup, 1856a; Steenstrup, 1856b; Steenstrup, 1857) added an argument for the taxonomic relevance of this “essential deviation from the symmetrical structure” (Steenstrup, 1857: p. 79), due to its species- and sex-specific location, and its (now undeniable) role in the reproduction of the animal.

As to the phenomenon of regeneration, Steenstrup emphasised its specificity to octopoda, which “*possess this power in the highest degree*”. “All the Decapoda”, on the contrary, “[appeared] to be incapable of replacing accidental injuries of the arms, or the loss of parts of them, by a new growth” (Steenstrup, 1857: p. 107). This was to prove a long-standing myth in the field of cephalopod regeneration studies, despite the numerous testimonies to the contrary (for a review see Imperadore and Fiorito, 2018).

In the natural-historical debate sketched here, regeneration of cephalopod appendages emerges as a peripheral, but important element in the characterisation of taxa, structures and modes of life, in close relation with sexual dimorphism, as well as sexual and “defensive” autotomy (on the categorisation of autotomy, *cf.* Stasek, 1967). Steenstrup, like Vérany, Vogt and Müller, put on the scientific record the fact of arm regeneration in cephalopods, which before was a matter of common experience.

He also added a remark, as an agenda for future investigators:

“I must content myself with having pointed out generally all the formations and agreements here described, and leaving it to those who possess richer materials, and especially to naturalists living on the sea-coasts, particularly that of the Mediterranean, who are fortunate enough to observe these animals daily in a state of nature, to carry out the comparison in all its details” (Steenstrup, 1857: p. 106).

Such a plea came from an authority in natural history, with three museums at his disposal (Steenstrup, 1857, note *: p. 83). Similarly, Vérany’s research critically depended on his institutional position, with all the connections it entailed, and the informal knowledge they contributed. Finally, the meaning of Müller’s decisive input (the observation of sex-specific traits in the argonaut male) had emerged against the backdrop of a close interaction between the locally-connected Vérany and the German colleagues.

### The octopus in a box—Marine stations, regeneration and cephalopods

Only 3 years after Steenstrup’s plea, the first European marine station was founded, at Concarneau (in 1859), on the Atlantic coast of France (Caullery, 1950). By 1900, there were more than sixty stations throughout the world (Dayrat, 2016), arguably an ideal infrastructure for pursuing Steenstrup’s programme. By the end of the XIX century, however, Steenstrup’s comparative-morphological approach had been superseded by a decidedly experimental one, with marine stations such as those of Naples (Italy) and Woods Hole (United States) playing a central role in the shift (Allen, 1975). On the one hand, regeneration became ever more firmly entrenched in a developmental framework, which entailed a focus on general “molecular” mechanisms (in animals, plants, and even crystals. Cf. Morgan, 1901) and a preference for simpler models, like the sea urchin embryo or the starfish, in addition to the traditional ones (e.g. salamanders and hydras; *cf.* Churchill, 1991). On the other hand, seashore laboratories contributed to the growing popularity of cephalopods mostly as physiological models, thanks to a level of organisation comparable to that of vertebrates (especially the closed circulatory system, unique among invertebrates, the complex nervous system, *etc.* Cf. Steiner, 1898), their tolerance to surgery and the remarkable viability of the explanted organs (Grimpe, 1928). In 1909, Bauer announced that “inkfish, and especially octopodes [were] about to rival frogs and rabbits” as physiological models (Bauer, 1909: p. 150). Just 2 years before, in his review of regeneration in the animal kingdom, Hans Przibram had remarked that knowledge of regeneration in cephalopods was limited to observational evidence, mentioning only Riggenbach’s, 1901 work on autotomy in *O. defilippii* (accepted name *Macrotritopus defilippi* Vérany, 1851) (Riggenbach, 1901) as the only experience with a bearing on the problem, under controlled conditions (Przibram, 1909: p. 130). It would indeed take the best part of a decade for a young scholar, by the name of Mathilde Margarethe Lange to devise the first systematic investigation of cephalopod regeneration in “standardised” conditions.

Lange was especially qualified for the task. Since 1910, she had read Zoology at Leipzig, Freiburg i. B, and Jena, attending the courses of the teuthologists Carl Chun (her first doctoral advisor), and Georg Grimpe. At Zurich, where she moved in 1917, she was supervised by Karl Hescheler, and attended the lectures of Adolf Naef, the authority in cephalopod systematics.

Lange experimented on live *O. vulgaris*, *Eledone moschata* and *Sepia officinalis*, at the Naples Zoological Station (in 1914), and the Musée Océanographique of Monaco (in 1915), providing macro- and microscopical description of all the stages of the process (cicatrisation, de- and regeneration), drawing comparisons between regeneration and embryonic development in cephalopods, and with the current results in invertebrates and vertebrates.

Cytological investigation yielded challenging results, especially as regarded the crucial mechanism of blastema formation. Since the 1880s, several competing theories of blastema formation had been proposed (Liversage, 1991). The prevailing one, named “epimorphosis” by Morgan (1901), had it derive from the dedifferentiation of neurones and muscle cells. These de-differentiated cells constituted the initial mass of the blastema, divided mitotically and re-differentiated returning to their original identity. What Lange observed in the octopus was instead a “double blastema”, as she named it. The “primary blastema” appeared to derive from the leucocytes carried by the blood vessels to the site of injury, where they phagocytised the cellular debris and formed the protective scar by agglutination (as cephalopod blood does not contain fibrin). After the regenerating skin had covered the site, the leucocytes appeared to transform, perhaps directly, into fibrocytes, the units of connective tissue. Lange’s “secondary blastema” (what we would today regard as the proper one) only began to appear after two or more days, displacing the primary without mixing with it.

Hescheler, Lange’s supervisor, was especially critical of her hypothesis of a direct transformation of one cellular type into another, as he made clear in his assessment of the dissertation^1^. The US zoologist, however, was unshaken by this opposition and concluded that the dermal connective constituted an exception to the accepted view that like tissues derive from like precursors.

As the differentiation process was concerned, Lange remarked how it was directly dependent on the contact with the regrowing tip of central axons, thus confirming the regulative role of nerves in regeneration, another hotly debated topic at the time (*cf.* Reiß, 2022).

By the time her dissertation appeared on the Journal of Experimental Zoology, Lange had moved back for good to the United States, where she made a career as Professor of Biology at Wheaton College (a women’s college in Massachusetts. McCoy, 2016, 139ff). She returned to Naples only once (November 1927-May 1928, at the American Women’s Table), to pursue further research on cephalopods, but no information is available either at the Zoological Station Archive, nor at Wheaton College about the activities she conducted during this visit^2^.

Surely, her first, ground-breaking stint had left open fronts. She had not followed the regeneration of suckers, passingly mentioned that of the eye lens, and only just raised the possibility of a different regeneration mechanism for the tip of the arm, where she had observed a permanent reservoir of undifferentiated embryonic cells. Finally, she had not really pursued a comparison between octopods and decapods, despite the general title of her dissertation (referring to the “arms of cephalopods”). Cuttlefish, Lange admitted, had proven too difficult to keep long enough. Nevertheless, she reported two intriguing cases of “compensatory regulation”, shown to her by Adolf Naef at Naples.

By the time Lange visited Naples for the first time, the Swiss zoologist Adolf Naef (1883-1949) was already well-known in the Station’s community, where he had been working since 1910 on a monograph on cephalopods for the series *Fauna und Flora des Golfes von Neapel*.

To Naef, the comparative study of the anatomy and embryology of a whole class afforded the possibility of an epistemological and methodological reassessment of morphological science, against two extremes: Haeckel’s phylogenetic morphology, with its emphasis on the recapitulation of developmental stages, and the excessive centrality of the phenotype and proximal causes preached by developmental mechanics (*cf.* Breidbach, 2003; Rieppel et al., 2013).

The debate between these two opposite positions had developed around the proper method for identifying homologies among organisms, and the very use of homology as a criterion in classification (*cf.* Laubichler, 2000). Since the early 1900s, the phenomenon of regeneration had taken centre stage in this debate. In a 1902 experiment, Hans Spemann and Hilde Mangold had extirpated the eye lens of a salamander embryo, and watched it regenerate completely, but from a different layer of tissue than its original precursor. This result disproved Haeckel’s theory of the Gastraea, a gastrula-like common progenitor of all animal forms (*cf.* Hoßfeld and Olsson, 2005). To Spemann, it also had wider consequences. If, as he argued in a later theoretical paper (Spemann, 1915), the regenerated lens had to be considered homologous to the extirpated one, then the very concept of homology had to be revised, and risked to lose most of its meaning. The problem was not only of explanatory frameworks, but also of methodology and approach: once accepted that ectopic regeneration was not an aberration, but true regeneration, then the proper way of elucidating the links between phylogeny and development was the study of the local conditions and mechanical processes that determined the phenomenon. This represented a complete reversal of Haeckel’s view on the relations between phylogeny and ontogeny, in which the latter became the basis for explaining the former. In methodological terms, this entailed the superiority of the experimental analysis of the mechanisms of development, over the systematic comparison of developmental stages.

Naef took an intermediate stand. On the one hand, he acknowledged the importance of *Entwicklungsmechanik* to morphology, and the criticism of Haeckel’s dogmatism. On the other hand, he found Spemann’s devaluation of homology too rush a conclusion to be drawn from a single experiment. To Naef, only a critical combination of all three approaches (comparative anatomy, plus descriptive and experimental embryology) could conclusively tell if regenerates of the kind observed by Spemann and Mangold were actually aberrations, or true homologies. The class Cephalopoda was of the right size for such an endeavour: large enough to allow empirical definition of homologies, but also small enough to be worked out by a single researcher, on the basis of a well-defined epistemic strategy. Comparative study of cephalopods held promise of yielding general concept of “type” and “typical stages” of development, based on the comparison between adult forms, to which he devoted the first volume of his work (Naef, 1972 [1921-1923]), and of developmental series of the greatest possible number of species (object of the second volume. Naef, 2000 [1928]: p. 342). Naef (1972) [1921-1923] Naef framed the phenomenon of regeneration as one element of a complex epistemological edifice, with the purpose of assessing the proper hierarchy of the different perspectives on morphology. To him, a science of form could only be founded on a comparative outlook, and the generalisation of results from single experiments, was misleading (Naef, 2000 [1928]: p. 342). Far from having consigned the problem of homology to the dustbin of history, experimental embryologists had to accept that an appropriate grasp on developmental mechanisms rested on a proper assessment of the relation between local, mechanical forces and typical, inherited developmental mechanisms (Naef, 2000 [1928]: p. 343). The brief experimental coda, attached to his great systematic effort, was meant to show just how this could be done.

In the succinct section two of the second volume (*On Disturbed and Abnormal Morphogenesis and Its Relation to Normal Development*), Naef built the case for cephalopods as a unifying model for morphology, by providing some hints on their proper use in the laboratory. The section opened with regeneration of the outer organs, followed by two parts on abnormal development (naturally occurring and experimentally induced). Naef noted the ubiquity of regeneration within the class (including, most clearly, arms and tentacles of decapods), the relative ease of obtaining it experimentally (Naef, 2000 [1928]: p. 343), and the possibility of contrasting several species-specific patterns of regeneration. He mentioned autotomy in *O. defilippii* (*M. defilippi*), as well as the interesting case of the loss of one dorsal arm in the argonaut, in which the remaining arm takes over the function of generation and repair of the shell. As Steenstrup had done before, Naef also warned of the possible misleading effect of arm regeneration on the identification of freshly caught specimens (p. 344).

If Naef’s coverage of regeneration in octopods was an orderly summary of the state of knowledge, the part on decapods offered new, first-hand observations, which he thought had potential for opening a few fronts of research. He noted that, apart from arms and tentacles, also small parts of the fin, arm membranes, eyelids and mantle regenerated easily, and that the phenomenon was easily controllable in the laboratory. Abnormal regenerates (heteromorphoses) were also often encountered in decapods, and in this connection Naef provided a lengthy description of the two extraordinary specimens mentioned by Lange in 1920. Probably because of the special position of the injuries, very close to the base of the arm, and to the buccal lappets, both specimens presented some mechanism of compensation (the “compensatory regulation” mentioned by Lange): the injured arms had not regrown, but in their stead, the corresponding buccal lappets had grown, slightly changed their position, fused with the injured stumps and started to develop suckers. The result was an intermediate condition between prehensile and buccal arms, confirmed by histological examination of their muscular connections. To Naef, the value of these exceptional instances was epistemic, in the first place. Sound knowledge of “the animal studied or developmental stage in all its details and […] multiple relationships with other members of the greater framework of order” (Naef, 2000 [1928]: p. 343), of the kind his monumental work had provided, allowed to determine whether these were cases of atavistic regeneration, or the expression of a “normally existing tendency” (p. 346). A firm experimental science of the mechanisms of adaptation, therefore, was critically dependent on the distinction between typical and atypical phenomena, which could only be rooted in comparison.

Naef intended to publish a more detailed study on the two cuttlefish specimens, but this promise, to the best of our knowledge, remained unfulfilled. He was never to see the Naples Station again, after his last 10-month visit in 1926 to complete the volume, and never to return to cephalopods (*cf.* Boletzky, 1999; Rieppel et al., 2013).

In their diversity, Lange and Naef’s takes on cephalopods as models for regeneration studies nicely complement each other. The former broke the ground for an experimental study and mechanistic interpretation of appendage regeneration in a so-far neglected animal class. The latter tried to reconcile two apparently opposing epistemic stands, by fashioning cephalopods as research models allowing the convergence of the comparative-anatomical and experimental-physiological approaches to morphology. Yet, both conspicuously failed to make any impact on contemporary regeneration research.

Lange’s dissertation was published in 1920, in the *Journal of Experimental Zoology*, which counted among its editors the US authorities on regeneration: Ross G. Harrison, Jacques Loeb, and Thomas H. Morgan. None of them seemed to take notice, however, for their way of framing regeneration was different. Although all of them researched on a variety of organisms, they did so mostly on account of the experimental advantages these offered towards a general physico-chemical, or at least mechanistic interpretation, rather than in a traditional comparative spirit. As Loeb put it in 1924, “We are already in possession of a number of enigmatic though often interesting observations on regeneration”, relic of a “stage of blind empiricism”, which made it difficult to discern whether one was getting lost in “a jungle of futile experiments”. What was needed, instead, were models amenable to precise quantitative work (Loeb, 1924: vi-vii), or well-chosen examples of generalizable mechanisms (Cf. Maienschein, 1991; Maienschein, 2010, on Harrison; Sunderland, 2010 on Morgan). The comparative approach loosely informing Lange’s study, and the interesting peculiarities she highlighted, were not what the US-American masters of the field cherished most. Nor did their European counterparts, reared in the same experimental-embryological tradition (*cf.* Barfurth, 1923; Przibram, 1926).

The fate of Naef’s synthesis is more nuanced. His *Fauna und Flora* monograph was saluted upon appearance as “the Bible of Theutologists” (Boletzky, 1999), and his epistemological stance was taken seriously and developed by a number of German-speaking scholars (from Adolf Portmann to Willi Henning), eventually constituting one pillar of the cladistics approach in the 1950s (Williams and Ebach, 2008). Yet, his ecumenical program for comparative and experimental embryology, centred on cephalopod regeneration, went completely unnoticed, as it fell in-between different audiences. On the side of systematics, the rise of the Evolutionary Synthesis, between the 1930s and the 1950s (Huxley, 1942), marked a disciplinary shift, consolidating around a nexus between the genetic, palaeontological and populational approaches, at the expense of the developmental. Despite occasional attempts of “translation” and introduction to Anglophone audiences (e.g. Zangerl, 1948), systematic morphology was actively side lined by the leaders of the Synthesis as a rear-guard approach (*cf.* Williams and Ebach, 2008: p. 62-63): Naef’s works were only translated into English from the 1970s (Naef, 1972 [1921-1923]).

As for the morphological disciplines of comparative anatomy and developmental mechanics, Naef’s call to collaboration, and his idea of cephalopod regeneration as a common field, also fell on sterile ground, because of the diverging paths of regeneration research, on the one side, and the perception of cephalopods as models, on the other side. On both shores of the Atlantic, regeneration was more than ever entrenched in an embryological framework, encompassing explanatory paradigms, methodology and the whole organisation of experimental systems, including animal models. Already before Mangold and Spemann’s spectacular demonstration of the “organiser effect” (Churchill, 1991: p. 116), and even more so after it (and Spemann’s 1935 Nobel Prize), the experimental object of choice for regeneration research were amphibians, especially urodeles. Apart from their very long association with regeneration since Spallanzani, salamanders and other germane species represented the perfect point of encounter between many different takes on regeneration. They afforded observation of normal and disturbed development at three different stages (embryo, larva, adult), and comparison among different species, which were not overly difficult to rear in captivity. Finally, and crucially, it was on such models that the practices of homo- and heteroplastic transplantation had been developed and perfected (what Reiß, 2022 calls “the practices of the cut and paste”). Cephalopods, on the contrary, raised many difficulties of management and interpretation. They were much harder to breed in captivity; their developmental stages were not as uniform and well understood as those of amphibians (Young and Harman, 1988), their taxonomy was constantly under revision, and even their age was extremely difficult to assess. Finally, such extreme experimental procedures were not possible, either because the animals were not resistant enough (this is the case for cuttlefish), or because those that were, like the octopus, presented peculiar problems: their arms could reach any part of the body, and boycott the recovery process (Boycott et al., 1965). A basic approach like Lange’s, or even the more refined one, only sketched by Naef, could not compete at the same level with Spemann’s experimental system. Moreover, the times of intense discussion of the evolutionary origin of the regeneration capacity (c.f. Goss, 1992) were long gone. Proximal causes and environmental influences were the name of the new game, and wide comparison across classes was a luxury that, perhaps, only a few, well equipped marine stations (like those of Naples or Woods Hole) could offer. Even there, knowledge of the material and methods for long term, comparative studies of regeneration were limited to a narrow circle of connoisseurs.

This is not to say that cephalopods had not consolidated their position as laboratory animals, on the contrary. A curious work, published in 1928 by Georg Grimpe, testifies to the growing demand of cephalopods as physiological and zoological models. A chapter of Emil Abderhalden’s encyclopaedic “Handbook of biological work-methods” (*Handbuch der biologischen Arbeitsmethoden* 1911-1939. Cf. Grote, 2018; De Sio et al., 2020, suppl. mat.) bore the title *Pflege, Behandlung und Zucht der Cephalopoden für Zoologische und Physiologische Zwecke* (“Care, Treatment and Rearing of Cephalopods for Zoological and Physiological Purposes”). What is revealing of this highly technical precis on methods and techniques is its focus on the demands of inland research aquaria—a sign of the growing fame of these “marine Guinea-pigs”, as he called them (Grimpe, 1928). Pupil and successor of Chun, Grimpe was a frequent guest of the Naples station and of many others, and could rely on the wisdom of the greatest teuthologists of the time. In fact, a great share of the technical information conveyed by Grimpe came from personal experience, or personal communication, but the overall picture he painted was one of great progress, especially in prolonging the survival of both captive octopods and decapods. Significantly, the concluding section (Grimpe, 1928: pp. 388-402), was devoted to the rearing of animals from the egg, a feat that had been tried with varying success since the 1880s (c.f. Joubin, 1888; Gravely, 1908; Drew, 1911) and to which Naef (1928) had attached a great importance as a means for turning cephalopods into the connecting link between systematic and experimental approaches. Although the rearing techniques for cephalopods (especially octopods) were nowhere near the level of development necessary for competing with amphibians or echinoderms in embryological studies, Grimpe’s summary conveyed the hope that, with a wider, planned effort, the difficulties could be overcome. In this voluminous chapter (mostly focussed on the common Mediterranean species), however, regeneration appears only marginally, and mostly in connection with the care of the animals. Lange’s procedures are duly described, and there is mention of regeneration of the eye lens, as well as of autotomy in *O. defilippii* (*M. defilippi*), but no treatment of regeneration experiments is provided, comparable to the much-better developed descriptions of physiological and psychological experimental systems. Moreover, Grimpe fell victim to the same misinterpretation of decapod regeneration as Lange. Although he gratefully listed Naef among his confidential sources, Grimpe (1928) bluntly stated that “no reliable proof of a natural regeneration has yet been adduced”, and, therefore “that Sepia, and even the more so the other decapods, are not suitable for experiments of this kind”.

The public Grimpe addressed had mostly other uses for cephalopods in its mind. Throughout the first half of the XX Century (Ponte et al., 2013) the greatest use of such animal models was in the field of neurophysiology, especially by means of chemical and electrical stimulation. Indeed, Grimpe reproduced almost the same list of experimental advantages as that proposed almost 20 years before by Bauer (see above). From the late 1920s, a new, productive front of investigation was opened, on the physiology, pharmacology and biochemistry of hormones and neurotransmitters (Bacq and Mazza, 1935; Erspamer and Boretti, 1951; Axelrod and Saavedra, 1977). Moreover, two pioneering experiences, by the Dutch animal psychologists Johannes A. Bierens de Haan and Frederik J. J. Buytendijk (academically “born” a physiologist) inaugurated the experimental study of octopus behaviour and of its neural underpinnings, a field that was to witness a great expansion after World War II (Bierens de Haan, 1926; Buytendijk, 1933).

### The 1930s

#### The Cajalian octopus

What, then, happened to regeneration research on cephalopods? Not much: in the roughly two decades following Lange’s publication, only three experimental works touching upon the issue appeared, with very little echo in the wider field (Sereni and Young, 1932; May, 1933; Callan, 1940). Out of the three, only May (1933) openly declared a link to Lange. Raoul Michel May had gained a PhD in Zoology at Harvard in 1924 with Samuel Detwiler, before moving to Paris, at the *Laboratoire d’Évolution des Êtres Organisés* (Ramón y Cajal et al., 1991). That same year, or in 1925, May spent some time in Santiago Ramon y Cajal’s laboratory in Madrid. As a consequence, he undertook the translation of *Degeneración y Regeneración de los Nervios* and *Degeneración y Regeneración de los Centros Nerviosos* (Ramón y Cajal, 1913; Ramón y Cajal, 1914), and started engaging experimentally with Cajal’s neurotropic hypothesis in which the Spaniard postulated the release of a chemical signal, emitted by the correlated sensory organ or the degenerating distal nerve stump, in order to account for the capacity of the regenerating peripheral axons of finding its regular path despite occasional detours. May (1925) first chose the catfish barbels as a test ground for the hypothesis, but the results obtained went in the opposite direction: it was the presence of the nerve that triggered the regeneration of the sensory organ. Between 1932 and 1933, he visited the Zoological Station of Salammbô (in the Regence de Tunis, a French protectorate at the time), where he seized the chance to try similar experiments on the suckers of *O. vulgaris* (May, 1933), which Lange had not followed in detail. Working on 11 specimens, May observed regeneration of the suckers after about 1 month and a half from the amputation of the arm. Histological inspection provided conclusive (and beautifully illustrated) anatomical evidence that the new suckers regenerate “absolument vierges d’innervation” in the epithelium and in the muscle. “We can count the cephalopod suckers”, he concluded, “among the organs that, functioning as guide and centre of attraction in the neurogenesis of their axon terminals (which do not seem to have a pre-established growth path), lend support to Cajal’s neurotropic theory” (May, 1933: p. 14, our translation). The octopus, it seems, was a fully Cajalian animal, much more so than the catfish, at any rate. The limited purchase of this study, and its publication in a rather obscure journal (the *Annales* of the Salammbô Station) conjured in keeping it unrecognized. Despite May’s effort, the neurotropic hypothesis had to wait about a decade for its final vindication: at the time, it was openly discarded by the authorities in the field (*cf.* Sereni and Young, 1932; Young, 1942; Weiss, 1944; Brauckmann, 2004).

Among the works taking a clear stance against neurotropism in axon regeneration, one (Sereni and Young, 1932) is of special interest here, as it was a study of cephalopod de- and regeneration. It stemmed from a collaboration, started in 1928, between Enrico Sereni, then head of the Physiological Laboratory of the Naples Zoological Station, and the British zoologist John Zachary Young. The latter had come to Naples in September 1928, to study the anatomy of the sympathetic nerves of fish. The encounter with Sereni changed his life: he chose to remain for a full year (instead of the 3 months originally planned), returned for eight more months between 1930 and 1931, and devoted the rest of his career to cephalopods.

**FIGURE 2:**
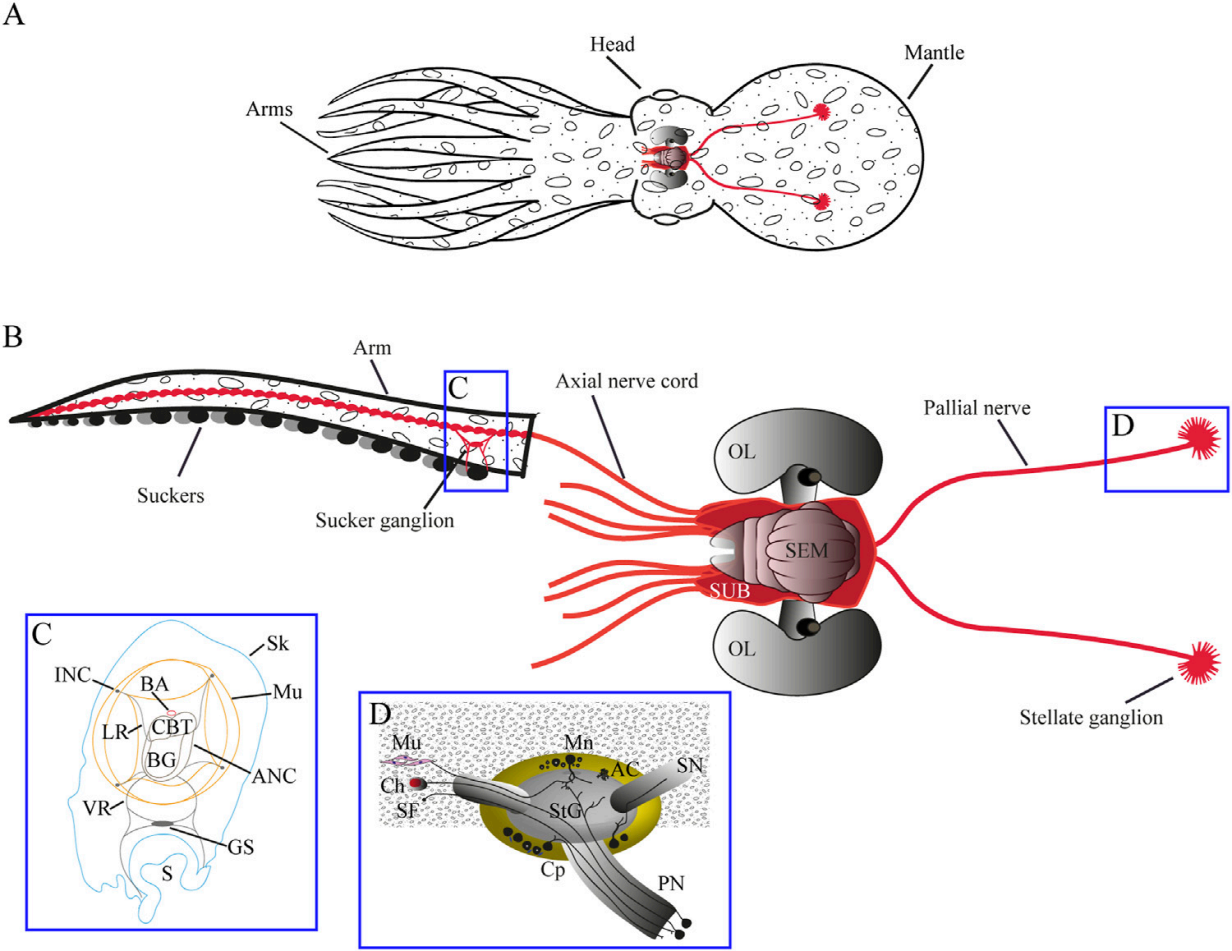
**(A)** Schematic drawing of *Octopus vulgaris* morphology. General anatomy is shown in **(A)** while **(B)** shows main structures of the nervous system with the brain (CNS) located in the head of the octopus, two pallial nerves arising (in red) from its posterior part and eight nerve cords (in red) from the anterior part innervating the arms. **(C)** highlights main structures in the arm (transverse section) and **(D)** highlights neural components of the pallial nerve and stellate ganglion, together with main connections. Particularly, pallial nerves are a paired neural structure composed of fibers covered in connective tissue, whose cell soma are mainly located in the subesophageal mass of the brain. Some of these fibers make synapsis **(D)** in the stellate ganglion for the control of the breathing muscles, while other axons travel directly to the skin to innervate chromatophores in the mantle (Young, 1971; Budelmann and Young, 1985). While complete transection of both nerves leads to animal death due to paralysis of respiratory muscles (Fredericq, 1878), the lesion of just one of them is easily managed by the animals, even though camouflage and breathing are impaired on the ipsilateral side of the injury (Fredericq, 1878; Sereni, 1929b; Imperadore et al., 2017). AC Amacrine cells, ANC axial nerve cord, BA brachial artery, BG brachial ganglion, Ch chromatophores, CBT cerebro-brachial tracts, CL cellular layer, CNS central nervous system, Cp centripetal cell, GS ganglion of sucker, INC. intramuscular nerve cords, LR lateral roots, Mn motoneurons, Mu muscular tissue, Nb neurobiotin, Np neuropil, OL optic lobe, PN pallial nerve, S sucker, SEM supra-esophageal mass, SF sensory fibers, Sk skin, SN stellar nerve, StG stellate ganglion, SUB sub-esophageal mass, v blood vessels, VR ventral roots. Adapted by permission from Springer Nature: Springer -Verlag GmbH Germany, Invertebrate Neuroscience: Neural pathways in the pallial nerve and arm nerve cord revealed by neurobiotin backfilling in the cephalopod mollusk *Octopus vulgaris*, Imperadore et al., Copyright ^©^ 2019.

Since 1925, Sereni had started a systematic study of the physiology of nerves, glands and chromatophores of cephalopods, and had succeeded in transferring to these molluscs some of the techniques developed on vertebrates (Sereni, 1929a; De Leo, 2008). His collaboration with Young, on the physiology and histology of the mantle connective (now pallial nerve), the stellate ganglion and the stellar nerves (see Figure 2 for details), was aimed at gaining a more precise functional topology of the nervous system of cephalopods. They did it by following regeneration after section or crushing of the nerves. The two published short communications on degeneration of the mantle connective already in 1929 (Sereni, 1929b; Young, 1929), and kept working on it until Sereni’s untimely death (De Leo, 2008). The task of completing the manuscript fell on Young only, who had unrestricted access to the histological material, as well as to Sereni’s notes (Sereni and Young, 1932).

**FIGURE 3:**
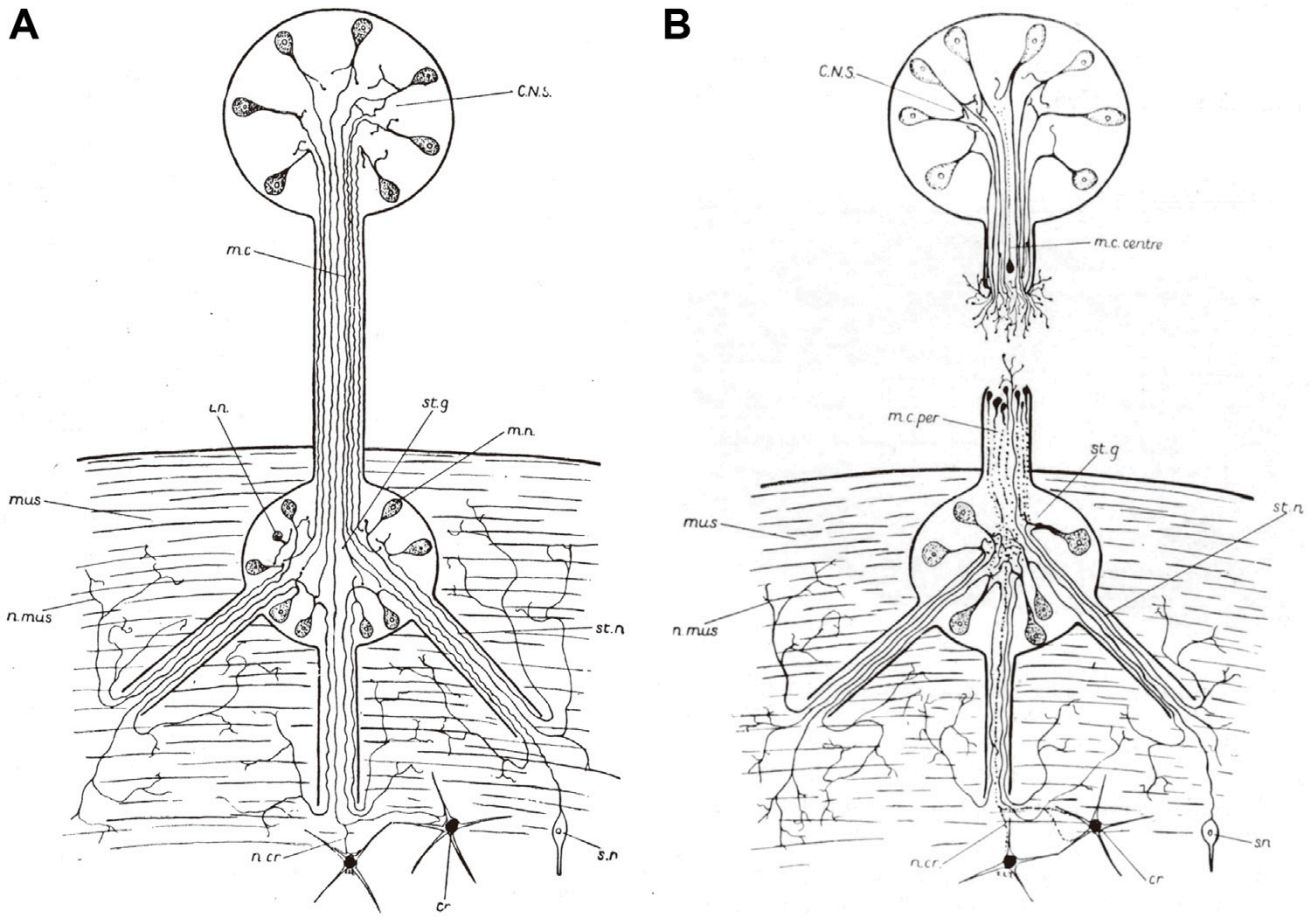
Diagrammatic drawings of pallial nerve. **(A)** Intact nerve. *CNS*, central nervous system; *m. c.*, pallial nerve (mantel connective, in the old terminology); *i n.*, intercalary neuron; *st. g.*, stellate ganglion; *m. n.*, motor neuron; *mus.*, mantel muscles; *n. mus.*, nerves to mantle; *st. n.*, stellar nerve; *n. cr.*, nerves to chromatophores; *s. n.*, sensory neuron. **(B)** Sectioned nerve in the process of regenerating. *m. c. centre*, central stump; *m. c. per.*, peripheral stump. Other lettering as in A. (Sereni and Young, 1932. Figure 1, p. 176, and 21, p. 195, respectively. ^©^ Stazione Zoologica Anton Dohrn. Reproduced by permission).

The material bases of the study were unprecedented: more than 200 specimens of different species, including decapods (Sereni and Young, 1932). Young framed it as a continuation of the work of Cajal and his pupils (Sereni and Young, 1932), of special importance because of the reliance of anti-neuronist theories on invertebrate models. Young reiterated that cephalopod neurons did not show any neurofibrillary continuity across the synapse: they were perfectly comparable to those of vertebrates, as of structure, responses to injury, and rate of axonal regrowth. As mentioned above, Young’s cephalopods were not as completely Cajalian as those of May: Young, in fact underscored the unorderly paths followed by regenerating axons, without any evidence of orderly directions and argued that re-growing axons probably followed the lines of least resistance (Figure 3). The physiological part of the work was scantier. At the time, Young had neither the interest nor the expertise for going into the minute detail, and mostly confirmed older results: the comparability of the mechanism in cephalopods and vertebrates, the decisive role of temperature and the central nervous control of chromatophores. One page, at the very end, reported, for the first time in any cephalopod, six cases of complete functional regeneration of the mantle connective. In four of them (all *O. vulgaris*, who survived between 110 and 140 days following surgery), the process of functional restitution could even be followed *in vivo* (Sereni and Young, 1932: pp. 204-205).

There was no follow-up to this report for about 40 years (Sanders and Young, 1974). Sereni’s passing was arguably a decisive factor in the interruption of regeneration research on cephalopods at Naples, as suggested by the last published work on the subject, a short note by H. G. Callan in the *Pubblicazioni della Stazione Zoologica di Napoli* (Callan, 1940). The experiment was based on Sereni’s own notes, made available to the author by Young. The simple procedure (extirpation of the gonads and observation of its effects on the regeneration of a lesioned hectocotylus) was the replication of one performed by the Italian physiologist in 1929 (Sereni, 1929a). The conclusions (absence of hormonal influence on hectocotylus regeneration) were at variance with the original ones, but, Callan reported, in line with Sereni’s later opinions as expressed in his notebooks.

After the war, Callan became a distinguished geneticist and cytologist at Edinburgh and St. Andrews. He remained a frequent visitor and protector of the Naples Station, but never resumed research on either cephalopods or regeneration. Young’s story is perhaps better known (at least in the field of the Neurosciences. Cf. Boycott, 1998) but is worth a short summary, as he was partly responsible for the long oblivion of cephalopod regeneration. His collaboration with Sereni famously led him to the re-discovery, in the early 1930s, of the squid giant axon (Young, 1985), which grew into the cornerstone of the biophysics of nervous transmission after World War II (Maxson, 2021). During the war, he was involved in what has been described as an example of translational neuroscience *ante litteram* (Lichtman and Sanes, 2006): the assessment of the regeneration rate of vertebrate neurons, in the attempt at improving surgical intervention on damaged peripheral nerves (*cf.* Young, 1942). His return to Naples in 1945, fresh Professor of Anatomy at the University College London, coincided with his return to cephalopods. His interests, though, had shifted from the peripheral to the central nervous system, and the physiological bases of learning and memory. In the following three decades, he catalysed a research effort on almost any aspect of cephalopod anatomy, modes of life and behaviour (mostly focussed on, but by no means limited to, *O. vulgaris*). Given the breadth of his interests, his previous history, the number of collaborators and fellow travellers that he attracted to Naples and, not least, the enormous number of cephalopods sacrificed, the complete disregard for regeneration comes to the eye. A look at his magnum opus, *The Anatomy of the Nervous System of Octopus vulgaris* (Young, 1971), a book 20 years in the making, illustrates the point. In what is still the most detailed analysis of the functional anatomy of a single species, no mention of regeneration is to be found in the main text. Even the chapter on the arm, by the expert Pasquale Graziadei, fails to cite the work of either May or (more surprisingly) Lange. The only, cursory, recurrences of the term are in the captions illustrating histological sections of the central nervous system, where re-growing axons appear following brain lobes removal (Young, 1971).

If, on the one hand, Young’s discovery of the squid axon, and subsequent research program on octopus memory were crucial in shaping the perception of cephalopods internationally (as models of nerve and of brain. Young, 1964; Maxson, 2021), on the other hand, the field of regeneration studies was undergoing a massive reorientation in a clinical direction. As Bernice Grafstein has argued, the period between the late 1940s and the 1980s witnessed major shifts in terms of institutional organisation and research priorities, also thanks to the involvement of charities, as well as of patients and veterans’ organisations. It is in this period that other incipient models, like the lamprey, providing a better proxy to the regeneration mechanisms of the spinal cord, gained the upper hand (Grafstein, 2000). A further layer of complexity and promise was added to regeneration as a scientific problem and, once again, cephalopods could not easily fit the framework.

### The 1970s

Young only returned to the problem of octopus regeneration upon his retirement from academic life, in 1974, once again with an intriguing but solitary stint. He and Geoff Sanders (Sanders and Young, 1974) returned to the dynamics of pallial nerve regeneration on *O. vulgaris*, in a preliminary attempt at exploring the underlying physiological mechanisms. The landscape of regeneration research had changed dramatically in the four decades since Young’s last contribution to the field: new evidence (c.f. Gaze, 1970) had revealed the full complexity of neural development and regeneration, and the undeniable role of chemical signalling and an increasing number of growth factors in it. This evidence derived from studies on a variety of models: chick and frog embryos, *in vitro* cultures and fish. To Sanders and Young, the pallial nerve-system in octopods, once developed, could outclass all existing experimental systems: it allowed observation *in vivo* until completion of the regenerative process and each single animal afforded comparison of the operated vs. intact side of the mantle. Crucially, the pallial system combined a relative simplicity of access and intervention on the nerve, with a very refined “tool” for the quantitative assessment of regeneration: the rate of recovery of texture and colour-patterning in the skin. The variety and highly stereotyped character of both colour- and skin-patterns of octopus (*cf.* Borrelli et al., 2006) offered reliable external marks of the progress of regeneration.

Sanders and Young compared photographs of ca. 30 specimens after acclimatisation, and then at different stages of recovery after crushing, resection, or complete transection of the pallial nerve. Their conclusions were as intriguing as they were tentative, and raised baffling questions. In particular, five specimens showed “practically complete” recovery of chromatophore control, i.e., a “fully normal” pattern of response, as shown by comparison between the operated and un-operated side, and between pre-operation and post-recovery photographs (*cf.* Sanders and Young, 1974). How to account for such precise functional restitution in terms of the physiology of regeneration, however, remained mysterious. How could the colour- and skin-pattern changes be so faithfully restored? Having excluded the unlikely extremes of random re-innervation, and a total rewiring of the nervous system, Sanders and Young were left with the hypothesis that the regenerating axons reconnected “with their original end-organs” (p. 10), a mechanism about which, by their own admission, they remained “totally ignorant”. A personal communication by Andrew Packard was reported at the end of the article, pointing at some “degree of functional control of patterning within the skin”. This hypothesis helped at least to reduce the complexity of the phenomenon: instead of a one-to-one relation between nerve fibres and chromatophores, it posited that innervation may occur through a single axon reconnecting with all the chromatophores involved in a patterning component. Just how, exactly, the regenerating axon was supposed to find its way (either by guidance from the muscle fibres, or by reconnection to their “own labelled tubes”), was a matter for further research, exploiting the favourable experimental conditions offered by the system.

Neither Young, nor anyone of his entourage did follow up on this effort. In a number of studies published in the 1990s, Packard resumed experimentation on de- and regeneration of the pallial nerve, in the framework of his whole-animal investigations on the central control of chromatophores. There again, although regeneration as a phenomenon resurfaced in intriguing ways (see, e.g. Packard, 1991; 1995), regeneration as a problem, to be mechanistically accounted for, did not.

#### Decapod regeneration

The most systematic study of regeneration of the 1970s was, again, the almost single-handed work of a PhD student, the French Jean Pierre Féral, at the biological station of Roscoff. It stemmed from a comparative research programme started by Féral’s supervisor, Pierre-Marie Lenicque, at the *Laboratoire de Biologie des Invertébrés* of the *Museum National d’Histoire Naturelle*. A student of John Runnström, since the mid-1950s, Lenicque focussed on the induction and inhibition of development (c.f. Lenicque, 1959). Towards the end of the 1960s, he had turned his attention to the metabolism of biological amines, and their role in regeneration in a variety of marine invertebrates (c.f. Lenicque and Féral, 1977). Féral’s doctoral research (Féral, 1977) was intended to further this line of research on an invertebrate model, the cuttlefish, presenting a greater degree of complexity and allowing for the exploration of the distinctive roles played by the nervous and circulatory systems in regeneration. The high development of these systems in cephalopods (the only invertebrates endowed with a closed blood circulation) made them a good proxy for vertebrates in whole-animal studies, with the added advantage of a greater accessibility of their central nervous system.

The experiments were performed between 1975 and 1977 at Roscoff, where he could rely on the guidance of Katharina Mangold, co-leader of the teuthology research group of the Laboratoire Arago at Banyuls-sur-Mer (Allcock et al., 2015). The first step was to confirm the regeneration capacity of cuttlefish, so much debated in the previous hundred years: from a survey of the area around Roscoff, Féral found ca. 2%–3% of adults, and around 15% of juvenile cuttlefish with regenerating appendages. He also established experimentally that cuttlefish could regenerate their fin, but only if cut transversally, while a longitudinal section caused the death of the animals (Féral, 1977). Building upon the pioneering work of a bunch of German, French, Spanish and US-American researchers since the mid-1960s (c.f. Sykes et al., 2014; Hanlon, 1990), Féral also set up a system for rearing the animals in captivity for a complete life-cycle, including reproduction. Only very few specimens raised in captivity, however, were actually used for the experiments. Following Lange’s example, Féral provided a fine description of the structure and development of the cuttlefish arm, and a thorough macroscopic account of the different phases of regeneration (from cicatrisation to functional restoration) under controlled environmental conditions (temperature, food, age. Cf. Imperadore and Fiorito, 2018). The ensuing picture, in the main, overlapped almost perfectly with Lange’s results. At a cytological level, Féral confirmed the role of amoebocytes in cleaning the site of lesion from the remains of the degenerating tissues. He also minutely described their participation in the formation of both scar and blastema (to which they appeared to be the greatest contributors), but did not even mention Lange’s double blastema hypothesis. The structural analysis of the regenerating and control arms revealed a sudden peak in general collagen production from the third day, and then again at the end of the second week, indicative of a participation of the whole organism to the phases of cicatrisation and of blastema-growth. Finally, Féral provided a general account of the combined role of the epithelium, the nervous and the blood system in regulating de- and regeneration, in a picture comparable with the one provided by urodeles. The conclusions drawn from this part of the work were mostly tentative (c.f. also Féral, 1979), leaving a number of open questions. Whereas myoblasts and neuroblasts seemed (topographically) to recover their original nature, the amoebocytes, after de-differentiation (Lange’s primary blastema) appeared to re-differentiate into connective tissue cells and later, supposedly, into chromatophores and iridophores. Just how this whole process was regulated, and what parts were played by “messengers” such as neurotransmitters, or by direct cellular interactions, remained unclear. Moreover, questions persisted regarding the exact way in which the nervous system influenced the process (whether by the direct action of neurotransmitters secreted by the axonal tract of the arm, or indirectly, by neurosecretion), as well as concerning the relation of amoebocytes and fibroblasts (whether the former developed into the latter, or both derived from a common precursor). All these open questions were incorporated, in 1978, in the research project Féral proposed for a post of Attaché de recherche (research associate) at the CNRS. Despite his dissertation having obtained highest honour by the university, the CNRS commission showed a distinct distrust for cuttlefish as a model for regeneration (Féral, personal communication) pushing Féral toward a distinguished career in a different field (evolutionary molecular biology and ecology) within the CNRS. Cuttlefish, on the other hand, underwent a 15 years-long eclipse as an experimental model for regeneration (Hielscher et al., 1996; Rohrbach and Schmidtberg, 2006), once again made more conspicuous by a parallel wave of popularity of cephalopods worldwide.

Since the late 1970s, two major initiatives had shaped the landscape of cephalopod research in different directions: the National Resource Center for Cephalopods, created in 1975 by Roger Hanlon (then at the University of Texas), and the Cephalopod International Advisory Council (CIAC), born of the initiative of a small community of cephalopod researchers in 1983 (Hochberg and Hatfield, 2002). Hanlon and collaborators sought to exploit the growing popularity of squid giant axons in biomedical research, in order to promote a wider range of cephalopod models in the field (e.g., Hanlon, 1990). The CIAC, instead, coalesced around interests in the systematics, ecology, behaviour, embryology, parasitology, physiology, and culture of cephalopods, and sought a programmatic connection with the fisheries sector. Once again, regeneration research fell somewhat in-between the focuses of these two initiatives, not absent, but nowhere near the core.

## Cephalopod regeneration today

The experimental study of regeneration in cephalopods was only revived from the end of the XX century, starting from where it had been left in the 1970s. Despite the general awareness of the width of cephalopods’ regeneration capacities (muscles, cornea, fins, peripheral nerves, CNS, etc), the arms and pallial nerve have remained the targets of choice in regeneration studies, with a major focus on two species, *S. officinalis* and *O. vulgaris*, out of nearly 800 (for review see Table 1 in Imperadore and Fiorito, 2018). The restricted choice of organisms to study depends on the abundant availability of experimental data for the above-mentioned species and on the so far limited number of observations and experimental capability possible on other species (consider for instance the case of *S. pharaonis* in Tressler et al., 2014). The core of such investigations, so far, is unsurprisingly concerned with reassessing the previous results and trying to answer the many questions left open, with the aid of novel techniques and within renewed research frameworks. The progress made in designing, adapting and developing cutting edge methodologies and approaches for this taxon in recent times, allowed for the elucidation of the first cellular and molecular pathways involved, even though these discoveries are still in their infancy.

The behavioural changes accompanying induced autotomy in the wild and lab (Crook et al., 2011; Bush, 2012; Alupay et al., 2014), as well as surgical amputation, have been considered more systematically. Except in the case of major ablations (80%–90% of one arm in cuttlefish, Tressler et al., 2014), no signs of behavioural modifications were observed in deeply anesthetized subjects (Fossati et al., 2013; Shaw et al., 2016). Regardless of the setting used to induce arm loss, complete and functional regeneration was always observed, independent of amputation level, with the regrowing arm being able to reach the same length of its contralateral structure (Tressler et al., 2014).

A systematic evaluation of the frequency of arm injury in natural conditions has also been attempted, yielding figures between 26% and 70% (Voight, 1992; Florini et al., 2011; Voss and Mehta, 2021) depending on species and geographical areas.

The crucial issue of comparability between surgically induced lesions (by means of different sharp tools, producing clean transections, under anaesthesia and in sterile conditions) and naturally occurring traumata (resulting in irregular injuries in presence of other possible undefinable intervening factors) has been left unattended throughout the last century. This is understandable, since defining a reproducible lesion method, allowing for comparison within and among studies, under controlled conditions (water temperature, feeding regime, tank enrichment, etc) was a major issue. The different settings, however, may influence events, pathways, and mechanisms underlying healing and regeneration. How the problem of ecological validity can be profitably overcome, is suggested by a recent work on *O. vulgaris* (Imperadore et al., 2022). The study took advantage of the high incidence of damaged wild-caught animals for this species, reported to occur at a rate of around 51% in the Gulf of Naples (Florini et al., 2011) and apparently linked to sublethal predation, autophagy, mating and aggressive behaviours (see Imperadore and Fiorito, 2018) for testing label-free multiphoton microscopy in the investigation of regeneration in cephalopods (see below). Nevertheless, analysis of the imaged samples highlighted the involvement of similar stages, processes, tissues and cellular events, as described by Lange (1920) and Féral (1978), Féral (1979), and shows how lab and field studies of regeneration can be profitably combined to the advantage of research and ethical treatment of animals.

### Arm and pallial nerve *i. from lesion to recovery*

Recently, cephalopod arm wound healing was subjected to closer investigation (Shaw et al., 2016): wound closure was followed for the first 24 h after amputation in *O. vulgaris*, using classical histological staining, immunohistochemistry (IHC), high-resolution ultrasound imaging and electron microscopy. Despite the diverse experimental settings (water temperature, animals’ age, species, sex, surgical method, site of lesion) the newer studies (Tressler et al., 2014; Shaw et al., 2016; Imperadore et al., 2022) confirm the earlier macro- and microscopic accounts, and especially the key role of amoebocytes/hemocytes (Polglase et al., 1983; Imperadore, et al., 2022). Shaw et al. (2016) also suggested ***i.*** a role for muscles cells in plug development, ***ii.*** the involvement of apoptotic skin, muscle and nerve cells (assessed through the use of TUNEL Assay, for the identification of fragmented DNA) and ***iii.*** the hypothetical formation of a belt-like structure below the wound apparently functioning as an actin cable involved in a purse-string contraction.

The pallial nerve model has also been resumed after a 40 years-long eclipse (Imperadore et al., 2017; Imperadore et al., 2018; Imperadore et al., 2019a), with a wider scope. In connection with the recent, rising concern for cephalopod welfare, the more recent studies have expanded their focus to the behavioural responses to injury, as an index of the severity of the procedure. Soon after lesion and at recovery from anesthesia, a few animals exhibit intense grooming behaviour around the denervated mantle area, an action tending to last for a few hours after surgery; no other signs of pain or distress were ever observed (Imperadore, et al., 2017; Imperadore, et al., 2019a). In addition, evaluation of the animal welfare, assessed through the measurement of their predatory performance in terms of readiness to attack and type of attack (*e.g.,* Amodio et al., 2014; following Borrelli, 2007), highlighted no significant differences between the lesioned and control groups or among individuals of the lesioned groups before and after surgery (Imperadore et al., 2019a).

Another element neglected by previous studies is breathing resumption, a conspicuous and easily measurable index of regeneration (Imperadore et al., 2019a). Finally, the great difference in the time of recovery, as well as in the number of successfully regenerating individuals, between the old and new experiments catches the eye, although it is difficult to account for on the basis of the published accounts.

In the first account (Sereni and Young, 1932) 65 days were needed to identify earliest signs of chromatic functional recovery and muscle contraction (both incomplete), highlighted through electrical stimulation of the skin and of the stellate ganglion *post mortem*, with functional regeneration only observed during summer. In the experiment by Sanders and Young (1974), some animals never or only partially recovered the chromatic function; those described as ‘fully regenerated’, required a minimum time of 50–59 days in summer (23°C) after crush and 60–69 days in autumn (18°C) after nerve cut. Additionally, authors highlighted a mismatch between nerve regeneration and functional recovery.

The most recent work on the subject (Imperadore et al., 2019a), however, reports a remarkable 100% structural and functional regeneration, both during spring and autumn (water temperature between 18°C and 22°C). Particularly, while the timing for skin pattern recovery varied (fastest complete recovery at 45 days), the time required to observe regained control over papillae raising and breathing muscles contraction was set at around 1 month after lesion (30–37 days following surgery), independently of the temperature in all specimens. Possible causes of such a stunning difference may be the type of lesion performed, its localization on the nerve, or the different anaesthetics employed.

A detailed microscopical analysis of the events occurring after axotomy of the pallial nerve, confirmed the occurrence of Wallerian degeneration, where intense axon swelling, fragmentation and death is observed as a consequence of the separation from the soma (Imperadore et al., 2017, 2018). A few days after the trauma, fibres of the central stump start regenerating toward the *scar* tissue to penetrate it, while it requires much longer (around 2 weeks) to observe the same effect in the opposite stump (regenerating sensory neurones, Imperadore et al., 2017). Despite the disorganised appearance of the regenerating fibres, the two stumps direct regenerating fibres toward their end targets, eventually crossing the lesion site (Imperadore et al., 2017).

As regards the process of correct orientation of the regenerating fibres, the observations of (Imperadore et al., 2017; Imperadore et al., 2018; Imperadore et al., 2019a) lend support to Fèral’s hypothesis of the leading role of connective tissue, against Sanders and Young’s (1974) proposal of the “orientated strand of muscle” beneath the nerve as the means used by the fibres.

Finally, backfilling experiments on the regrowing nerve up to 5 months after lesion, are in agreement with Young and Sanders’ hypothesis of functional recovery though end-target reinnervation, with some fibres reconnecting to motoneurons in the stellate ganglion, and other crossing it to reach chromatophores at the periphery. However, although physiological and functional regeneration is achieved, the pallial nerve never restores its original structure, showing fibers aberration, swelling and branching even several months post lesion (Imperadore et al., 2019a). Unlike arm injury, pallial nerve regeneration remains a laboratory model, as no published account on the injury frequency in the wild for this structure is available.

### Arm and pallial nerve *ii. Cellular and molecular pathways*

Also Fèral’s open questions about the role of neurotransmitters in nerve regeneration have been resumed with the aid of more advanced techniques, with an indirect approach. Fossati et al. (2013); Fossati et al. (2015) measured the metabolism of acetylcholinesterase (AChE), the enzyme responsible for the breakdown of acetylcholine (ACh) in the regenerating arm of *O. vulgaris*. AChE activity, inversely correlated to ACh abundance, was found to drop during wound healing (3–17 days after damage) and reversed the trend only at the onset of regeneration (ca 18 days post lesion). In this instance, the active enzyme is restricted to the sole axial nerve cord. Return to activity basal level corresponds to complete morphological restoration, exactly like in *Triturus* (Singer et al., 1960).

A non-classical and non-cholinergic function was also suggested for AChE during arm morphogenesis in embryo development and in adult regeneration, again in *O. vulgaris* (Fossati et al., 2015). The enzyme was found to be expressed in non-neural regions, i.e., in the blastemal differentiating mesenchymal cells of the newly developing limb and in the blastemal structure that forms just after wound healing in the adult damaged arm, mainly composed of undifferentiated cells. These phases are characterized by intense cell proliferation. Mitotic cells appear diffuse in the whole early arm rudiment, later restricting to the most distal part of the tip as differentiation progresses (Nödl et al., 2015). Cell proliferation during adult arm regeneration appears to follow a similar pattern (Fossati et al., 2013), although Lange’s (1920) speculation on the “permanent reservoir of undifferentiated embryonic cells” at the tip of the adult arm (see above) remains to date unsubstantiated.

The characterization of HOX and Wnt genes in the regulation of development and regeneration (established for several metazoans: Ruddle et al., 1994; Petersen and Reddien, 2009; Holstein, 2012), is still at an incipient phase in cephalopods, as is that of the molecular fingerprint guiding and controlling arm growth and regeneration. The few available data are intriguing and encourage a specific attempt to pursue this research further.

Indeed, the expression patterns identified for HOX genes during embryo development in *Euprymna scolopes*, design a precise temporal and spatial distribution in some structures, suggesting a correlation between the localized gene expression and cephalopod morphological innovations (for instance, the funnel tube, the buccal crown or the light organs; Lee et al., 2003). Wnt proteins (Wnt1, Wnt2, Wnt5 and Wnt7), together with other molecules responsible for the regulation of proximodistal, anteroposterior, and dorsoventral axes, were instead proven active during limb development in cuttlefish embryos (*S. officinalis* and *S. bandensis*) and showed molecular regionalization, consistently with what observed in arthropods and vertebrates’ limb development (Tarazona et al., 2019).

Baldascino (2019) has explored the expression profile of about 30 genes in uninjured and regenerating octopus arms. Results reveal differential expression in the proximal arm areas, as compared to the tip. Moreover, some genes appeared up- and/or downregulated during different phases of arm regeneration (e.g., Wnt proteins, Hox-B7, Antennapedia; Baldascino, 2019).

In recent years, epigenetic regulation of gene expression during regenerative phenomena has become of great interest (for a review see Katsuyama and Paro, 2011), fuelled by the hope of finding ways to induce structural recovery in poor regenerators, such as humans (Barrero and Izpisua Belmonte, 2011). The questions of how these regulatory pathways work, and how they evolved, are still begging an answer, and investigation of cephalopods’ epigenome, among other regeneration competent organisms, may help filling some of these lacunas in a comparative perspective.

Evolutionarily conserved elements involved in DNA and histone methylation/acetylation were identified and found active in different tissues of *O. bimaculoides*, *E. berryi* and *Doryteuthis pealeii* (Macchi et al., 2022). Moreover, transcriptional analysis of control and regenerating structures in *O. vulgaris* highlighted dynamic gene expression profiles for some epigenetic regulators (Imperadore, 2017; Baldascino, 2019). In particular, the limb of adult individuals showed differential expression along its length, as for the case of the polycomb group (PcG) proteins of the PRC1 and PCR2 complexes, usually involved in the methylation of Histone H2 and H3, generally marking gene repression. These were found to be upregulated in the uninjured arm tip compared to medial and proximal arm areas, data also corroborated by gene expression analysis in another octopus species, *E. moscata* (Baldascino, 2019). A few genes of the same complexes, e.g., EZH2 and SUZ12 were also found upregulated during arm regeneration, particularly during blastema formation (Baldascino, 2019). It is worth noting that both, the adult arm tip and the regenerating blastema, are characterized by cells actively proliferating. As it happens for other species, PcG repressive marks may serve to induce or promote proliferation and allow for arm continuous growth and stump regeneration (Barrero and Izpisua Belmonte, 2011), although this remains speculation.

Although the summarised data appear to robustly support the commonality of developmental and regenerative pathways in cephalopods and other metazoans, we have to consider that a biased approach has been utilized so far. Unsurprisingly, research on cephalopods has until now replicated the pattern established on model organisms, relying on the available information from other species, and adapting the technology developed on them. Recently (Ritschard et al., 2019; Schmidbaur et al., 2022), class- and species-specific orphan genes have been identified and tentatively linked to the evolution of cephalopod morphological novelties. It is at least plausible that such novel, still uncharacterized genes could also be involved in regenerative processes, although this again remains conjectural. Alternatively, it is also possible that known conserved molecules have pleiotropic functions (Sánchez Alvarado, 2004) as was observed for Hox genes in *E. scolopes* (Lee et al., 2003) where these well-known conserved genes are expressed in novel structures, specific to the class Cephalopoda.

### What future for cephalopod research?

Interest in deciphering and characterizing the regeneration toolkit of competent organisms has recently been boosted by the emergence of the relatively new interdisciplinary field of tissue engineering and regenerative medicine (Polykandriotis et al., 2010; Berthiaume et al., 2011; Mehta and Singh, 2019). Crucial features, such as accessible genomic and molecular resources and tools, amenability to genetic manipulation, fast generation time and ease of maintenance in laboratory conditions, mainly restricted regenerative studies to a few well-established animal models (Mokalled and Poss, 2018; Mehta and Singh, 2019), leaving a variety of species unexplored. In some cases, these epistemic advantages prevailed over the most crucial aspect of high regenerative capacity, allowing poor regenerators, such as *Drosophila melanogaster*, *Caenorhabditis elegans* and *Mus musculus*, to take the lead in translational studies.

The accelerating methodological and technological spillover, together with the release of publicly available *Omic* datasets, and, not least, the cost-optimization of cutting-edge technologies, revamped the interest for still largely overlooked, proficient regenerators, determining the possibility to elucidate common pathways as well as novel genes involved in the process (Sánchez Alvarado, 2004; Smith et al., 2011; Franco et al., 2013; Brockes and Gates, 2014; Casco-Robles et al., 2018). The release of the first cephalopod genome (*O. bimaculoides*, Albertin et al., 2015) set the ground for a new era: in less than a decade, the genome and transcriptomes of more than ten species have been published, together with chromatin profiling and mass-spectrometry datasets, for some. The enormous flow of new data highlighted some unique features of this class: extensive RNA editing, gene duplication, gene family expansion (e.g., GPCRs, Protocadherins, C2H2 ZNFs), large scale genome reorganization and emergence of novel genes. All these elements have been tentatively correlated with the organismal novelties identified in cuttlefish, squid and octopus (e.g., Liscovitch-Brauer et al., 2017; Ritschard et al., 2019; Schmidbaur et al., 2022).

The interest raised by these findings inspired deeper examination of cephalopod nervous system, the largest, most complex, and most cell-dense among invertebrates (Young, 1963; Grimaldi et al., 2007). A brain atlas (Deryckere et al., 2020; Deryckere et al., 2021), massive single-cell and single-nuclei datasets (Styfhals et al., 2022) were produced for *O. vulgaris* paralarval stages, allowing for novel insights into the characterization of molecular signatures of brain cells at early stage of development for the first time in a mollusc. A measure of the effort required, however, is given by the consideration that only for 9% of the 200,000 brain cells estimated in an octopus brain at hatching (compared to the 200 million in the adult), a single-cell expression profile could be obtained.

The possibility of altering gene expression *in vivo*, through loss and gain-of-function experiments, is a new standard in the study of regeneration. A range of genetic tools have been developed upon, and are currently employed in model organisms: RNA interference, transgenesis, chemical- and UV-induced mutagenesis, and, not least, CRISPR-CAS9 technology (Mehta and Singh, 2019), which, since its development in the 2010s, has held promise of connecting basic life science with biomedical and biotechnological applications. Cephalopod models have long been kept at the margin of this tumultuous development, due to the absence of these genetic tools. Very recently, however, Crawford et al. (2020) successfully applied CRISPR-CAS9 to squid embryos (*D. pealeii*) obtaining completely disrupted skin pigmentation: the first ever gene knockout in cephalopods.

Imaging regeneration has also proved advantageous, in several species, to investigate regeneration. Despite limited access to commercial markers or techniques for real time imaging, some tools have recently been developed: label-free multiphoton microscopy (Imperadore et al., 2018; Imperadore et al., 2022), 18F-FDG PET (Zullo et al., 2018), optimized CUBIC clearing protocol (Deryckere et al., 2020) and neural tracing (Imperadore et al., 2019a; Imperadore et al., 2019b).

## Conclusion

Here we overviewed a long journey of research around experimental systems—i.e., cephalopod arm and pallial nerve—and research questions together with intriguing, but always tentative, answers (Lange: “double blastema” and “reservoir of undifferentiated cells”; Féral: the role neurotransmitters in regeneration; May: chemical signalling in development and regeneration; Sanders and Young: the chromatophore control and its fate during regeneration). A final, recurring theme is peripherality, both as a limit (too little, too late), as well as a possibility.

Almost all of the older works we have considered, in fact, contain more or less direct suggestions of the specific contribution cephalopods could provide to regeneration research. Steenstrup - and, less directly, Féral - underscored the possibility of combining museum collections, sampling in the field and laboratory findings. Lange and Féral emphasized the intermediate position of the organisms: between the simpler invertebrates and the vertebrates. Naef vainly promoted cephalopod regeneration as a point of encounter between the opposed epistemic approaches of comparative anatomy and experimental embryology. Sanders and Young highlighted the experimental advantage of following neural regeneration in live subjects, as well as of having experiment and control combined in the same specimen.

Many of these suggestions have been taken seriously by present scholars in cephalopod regeneration. However, it is fair to say, the progress so far has consisted more in reformulating old questions and hypotheses in more contemporary terms, than in solving the issues (e.g., blastemal cell composition, cell positional memory, stem cell involvement, cell reprogramming, positional identity, dependence from the nervous system). A breakthrough in any of these research directions would arguably require a more intense and participated research effort, and a significant investment in time, expectations, and money. In this connection, the trivial historical question “why were cephalopods so peripheral?“, and the less trivial experimental one “what is needed to make them central?” conflate, and enlighten each other to some extent. Throughout the historical section of this paper, the concept of framework has resurfaced, mostly in considering the divergence between “frameworks of regeneration” and “frameworks of experimentation on cephalopods”. Here, “framework” must be read as the German “Gestalt”: the familiar perceptual complex that makes elements of a complex picture either stand out, or remain hidden. As we have seen, at different points in history a divergence has been stressed, between the framework of regeneration research (which includes not only how regeneration is approached, but also what it is considered to be), and the perception of cephalopods as experimental animals. Throughout the first half of the XX Century, regeneration was mostly framed in an experimental-embryological scheme, being considered as a proxy of developmental mechanisms. In the second half, this framework was supplemented, rather than replaced, by a translational one. This, on the one hand, enhanced the visibility of previously disregarded “models”, such as the lamprey (Maxson and Morgan—*submitted*). On the other, with its emphasis on harnessing the cellular-molecular mechanisms of regeneration, it has promoted other organisms such as *Drosophila* and mouse, not very proficient at regenerating, but closely involved in the development of critical technologies. In the meantime, cephalopods continued to grow in reputation as experimental models, just not of regeneration. Positioning as “marine Guinea pigs” within the framework of comparative physiology and biochemistry (Grimpe, 1928), the animals were later laboriously consolidated as experimental tools to explore neurone and axon, if not of the brain altogether. The intensive research activity undertaken in the past century allowed for the identification of many cephalopods’ special features, amongst which the complex behavioural and learning capabilities and the intricate and sophisticated nervous system, and the capacity to modulate behavioural responses elicited by stimuli considered potentially painful, stand out (Nixon and Young, 2003; Crook, 2021; Ponte et al., 2021; Ponte et al., 2022). The above-mentioned features supported the inclusion of cephalopods, as the sole invertebrate class, in the Directive 2010/63/EU (Andrews et al., 2013; Smith et al., 2013; Fiorito et al., 2015; Ponte et al., 2019; De Sio et al., 2020) regulating the use of animals in scientific research. Despite the original worries of creating disparities between regulated procedures applied to higher-vertebrates and cephalopods (Nosengo, 2011), this actually promoted a revived scientific attention for cephalopods, thus boosting current research effort (Albertin and Simakov, 2020). This brings us to the present situation, which encourages moderate hopes. Approaches and technologies developed for classic model organisms are spreading to other systems. Furthermore, the increased attention towards animal welfare and sentience of species to study (including cephalopods) is promoting a levelling-up of the ways to approach the study with non-model organisms, beyond legal obligations.

The technological advancement we are facing can open the way to a fresh start, and to the possibility of answering new, as well as old and long deferred questions. Regeneration is of course one of them. The emerging possibility of determining cephalopod gene function is exceptionally encouraging, especially considering the great number of genes with *de novo* origin not finding any similarity in other species (Ritschard et al., 2019; Schmidbaur et al., 2022).

This new horizon stimulates and requires choices, however, and strategies of persuasion. The second part of this review has shown that regeneration in cephalopods follows common steps with limb and peripheral nerve regeneration in vertebrates (e.g., Whited and Tabin, 2009; Simon and Tanaka, 2013), and that conserved pathways are most likely involved. On the other hand, it also strongly suggests that cephalopods could provide a suitable research object of genetic and epigenetic innovation mechanisms, adding another layer to the exploration of cellular and molecular machinery, i.e., the developmental—and more important—evolutionary and systems neuroscience perspectives.

No simple choice is available here, but a series of elements seem to be coming together into a coherent picture: model-organism-based biomedicine seems on the verge of becoming recent history, while the powerful instruments that were created in that context may prove decisive in overcoming its strictures, again towards wide comparative approaches.

It is at junctions like this, that daring choices by individual researchers are perhaps needed. On the other hand, no single researcher, and very few research groups, can afford spearheading a revolution that seems more plausible than probable.

Our ultimate goal is promoting further the investigation of cephalopods as organisms endowed with remarkable features, including what we can picture through the examination of their regenerative capacities. The phenomena occurring in these animals are plausibly leading to fascinating surprises, dubiously achievable with “classic model organisms” currently utilized in regeneration. Apart from the expectations linked to a gradual - but considerable - growth of new and cutting-edge experimental tools, which will offer new opportunities and challenges, we are fully convinced that cephalopods present unique and exciting opportunities, and the time might have finally come to take advantage of them.

## Footnotes

1 Hescheler, Karl, *Gutachten zur Dissertation „Beiträge zur Kenntnis der Regeneration und des feinenren Baues der Arme bei den Cephalopoden”*, 18.11.1919, Staatsarchiv Kanton Zürich, Signatur U 110.6.1505.

2 The Guest Researchers Database of the Station only records her presence in that period, with no mention of the subject. As for the Wheaton College Archives, they indeed keep all of Lange’s yearly reports, both as professor and as Dean. However, they are all limited to the didactical side of her activity, which speaks volumes about the consideration in which research was held in institutions of higher education for women at the time. In fact, Lange’s report for 1928 is entirely missing, and, according to McCoy (2016), she tried to finance her sabbatical by offering her services to the ONI again, since the College could not support non-teaching staff. Her offer did not elicit any reaction from the Office, so she arguably funded her research trip out of her own pocket (McCoy, 2016, p. 141).
